# Highlighting the importance of early diagnosis of cyclic vomiting syndrome in adults: A case report

**DOI:** 10.1097/MD.0000000000018365

**Published:** 2019-12-20

**Authors:** Cuilan Tang, Ning Dai

**Affiliations:** a Department of Infectious Diseases, The Second Affiliated Hospital, Zhejiang University School of Medicine, No. 88 Jiefang Road, Hangzhou 310009; b Department of Digestive Diseases, Sir Run Run Shaw Hospital (SRRSH), affiliated with the Zhejiang University School of Medicine, No. 3 East Qingchun Road, Hangzhou 310006, Zhejiang, China.

**Keywords:** adults, case report, cyclic vomiting syndrome, repeated vomiting

## Abstract

**Introduction:**

Cyclic vomiting syndrome (CVS) is a potentially exhausting disorder and has an adverse impact on quality of life, but it is poorly recognized and is always misdiagnosed leading to a diagnostic delay of several years, especially in adults.

**Patient concerns:**

We report a case of a 32-year-old woman with recurrent severe nausea, vomiting, and abdominal pain, and repeated visits to the emergency department or the outpatient department for 4 years. Each time she was diagnosed with gastroenteritis or gastritis, and recovered after supportive treatment including antiemetics, maintenance of water and electrolyte balance, and a proton pump inhibitor.

**Diagnosis:**

Laboratory examinations, gastroenteroscopy, chest and abdominal computed tomography, and brain magnetic resonance imaging all failed to reveal abnormalities that would explain her symptoms. Based on typical symptoms and the exclusion of other diseases associated with repeated vomiting, the diagnosis was made as CVS.

**Interventions:**

She was given orally amitriptyline, 50 mg per night, and olanzapine, 1.25 mg per night.

**Outcomes:**

The treatment was effective in inducing remission, and symptoms did not recur after treatment. The treatment lasted for 2 months and stopped. Her symptoms did not recur over the 10-month follow up.

**Conclusion:**

CVS is not rare in adults, but its diagnosis is usually delayed due to poor recognition of the condition. Clinician awareness of CVS should be enhanced to improve early diagnosis.

Core tip: Cyclic vomiting syndrome has a tremendous impact on the quality of life, but it is poorly recognized and is always misdiagnosed leading to a diagnostic delay of several years, especially in adults. The article presented a case report of cyclic vomiting syndrome of adult; we hope the article will attribute to increased awareness of physician and reduce delayed diagnosis.

## 1 Introduction

Cyclic vomiting syndrome (CVS) is a functional gastrointestinal disorder characterized by recurrent, stereotypical vomiting attacks, and normal intervals.^[1]^ CVS was first reported in children,^[2,3]^ and it was initially thought that the disease only affected children. However, it has been recognized in the past 20 to 30 years that this disorder can also be present in adults. Although the Rome IV criteria for the diagnosis of CVS in adults were established in 2016, the condition remains under-recognized, and CVS in adults is often misdiagnosed, leading to delayed treatment.^[4]^ Although this syndrome affects children and adults, adult CVS is less common. We here reported a case of a 32-year-old woman with recurrent severe nausea and vomiting in 4 years, and her symptoms did not recur after diagnosis and treatment of CVS. We also review the literature on CVS in adults.

## 2 Case presentation

The Ethics Committee of the Second Affiliated Hospital of Zhejiang University School of Medicine has approved the study. A 32-year-old woman presented to the outpatient department with recurrent severe nausea, vomiting, and abdominal pain for 4 years. The patient was a network engineer, she is married and has a 3-year-old son. She suffered from stereotypic episodes of nausea, vomiting, and abdominal pain lasting 2 to 3 days in 4 years. The attacks typically started in the morning with abdominal pain, and then nausea and vomiting (the stomach contents). No fever, night sweat, diarrhea, chest tightness, cough, sputum, dizziness, headache, muscle ache, or sweating occurred. She did not suffer from migraine. The episodes occurred approximately once every 2 or 3 months, but occurred frequently about once a month for half a year. There was no nausea and vomiting between the episodes. She has visited several hospitals (including top 100 hospitals in China) and was diagnosed with gastroenteritis or gastritis, and recovered after supportive treatment including antiemetics, maintenance of water and electrolyte balance, and a proton pump inhibitor. Several attacks occurred before menstruation, and the patient had been examined by a gynecologist who failed to find a disorder to explain her symptoms. In order to exclude suspected somatic and psychiatric comorbidity, she was examined at our department of psychiatry and a psychiatric diagnosis was excluded. Following another attack, she attended the hospital again. The patient had previously been healthy and had no past illnesses. Her family history, including migraine, was unremarkable.

On physical examination, the patient was 155 cm in height, body weight was 44 kg, and BMI was 18.3 kg/m^2^. Her blood pressure was 110/86 mm Hg and pulse rate was 75 bpm. She had clear lungs and normal heart sounds with no murmurs or gallops on auscultation. No gastrointestinal or peristalsis abnormalities were observed. Tenderness was obvious in the upper abdomen. There was no rebound tenderness in the abdominal or renal region. Liver and spleen were untouchable. There were no other positive signs.

Extensive examinations were performed, including routine blood test, C-reactive protein, liver function test including transaminases, lactate dehydrogenase, and total protein, pituitary hormones, creatinine, thyroid function tests including thyroxine and the thyroid-stimulating hormone. All results were within the reference range.

Imaging examinations including gastroenteroscopy, chest and abdominal computed tomography, and brain magnetic resonance imaging all failed to reveal abnormalities that would explain her symptoms.

Her symptoms reappeared and she was referred to our hospital 6 times in 4 months; each time she was diagnosed with chronic gastritis and was given supportive treatment including antiemetics (including Ondansetron), maintenance of water and electrolyte balance, and a proton pump inhibitor for 2 to 3 days; the treatment was effective, and she recovered and discharged from hospital, then followed with asymptomatic interval about 1 month. During the last hospitalization, she was diagnosed with CVS based on her medical history and the exclusion of other differential diagnoses according to the Rome IV criteria. Then she was given amitriptyline 50 mg and olanzapine 1.25 mg per night.

After the treatment, the attack stopped. The treatment lasted for 2 months with the same plan and stopped. The reason for stop was because the symptoms did not appear after the treatment and side effects such as dry mouth, although the side effects were mild and tolerable. Her symptoms did not recur over the 10-month follow up. During the last follow up, her mental state has improved, her weight has increased from 44 kg to 50 kg, and her BMI has increased from 18.3 to 20.8 kg/m^2^, reaching a normal value.

## 3 Discussion

CVS is a chronic, functional gastrointestinal disease. The incidence of CVS is low; adults typically develop CVS in middle age,^[5]^ the lack of characteristic markers leads to inadequate recognition by physicians, patients are often misdiagnosed as having recurrent gastroenteritis and food poisoning, resulting in a delayed diagnosis.^[6]^ Compared with other functional gastrointestinal diseases, CVS has been associated with more serious health damage^[6,7]^ and high rates of health care utilization.^[8,9]^

The pathogenesis of CVS is unknown, but it has been associated with migraine.^[10]^ CVS was also associated with mitochondrial dysfunction, autonomic nervous system dysfunction, activation of the hypothalamic-pituitary-adrenal axis, and menstruation.^[11–14]^ In addition, it is reported to be related to the long-term use of cannabis, with the characteristic of repetitive hot water bathing behavior during vomiting cycles.^[15,16]^

There are two essential features of CVS, one is stereotypical episodes of vomiting regarding onset (acute) and duration (hours to days), and the other is the absence of nausea and vomiting between episodes. The specific pattern of vomiting episodes is variable among patients but, importantly, is stereotypical for an individual patient.^[17]^ In most cases, vomiting attacks can be triggered by stress, fatigue, or the start of menstruation.^[18]^ The trigger of our patient was stress and tiredness, and the attack usually occurred before menstruation. It was reported that menstruation was the most frequent trigger in women.^[19]^ The asymptomatic period can last from weeks to months and is changeable among patients

The diagnosis of CVS is based on the history and exclusion of other diseases; the Rome IV criteria (Table 1) suggested that expensive and unnecessary investigations should be avoided,^[20]^ because the vast majority (90%) of patients with symptoms consistent with CVS did not have any organ abnormalities on investigation.^[4]^ Diseases requiring differential diagnosis in relation to CVS include peptic ulcer, pancreatitis, gastroparesis, intermittent small intestinal obstruction, appendicitis, renal colic, adrenal insufficiency, and central nervous system diseases,^[21]^ and most of these disorders can be excluded by careful history taking and minimal testing.

**Table 1:**
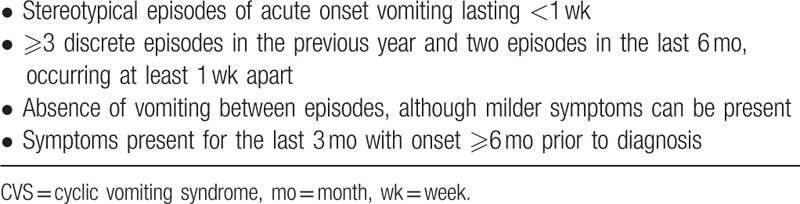
Adult Rome IV diagnostic criteria for CVS.

**Figure 1:**
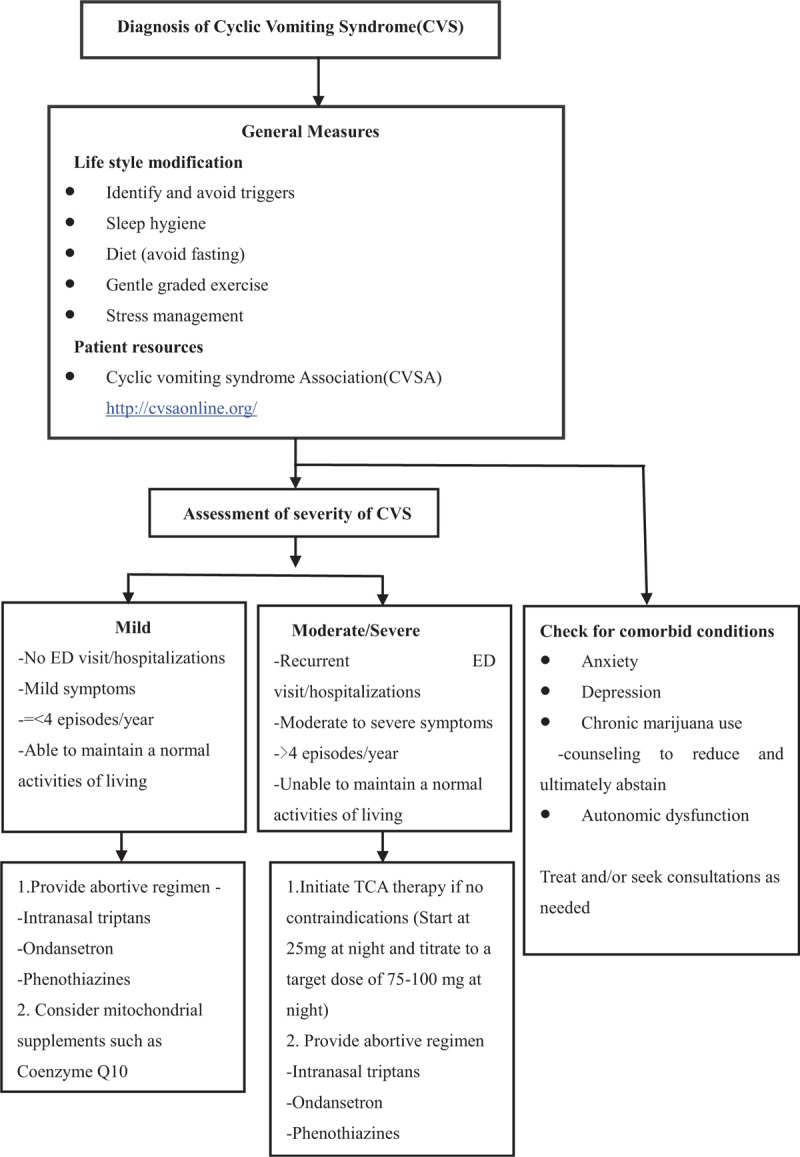
Suggested algorithm for management of cyclic vomiting syndrome.

Lifestyle modifications and trigger avoidance are essential for successful management. To date, no controlled treatment trial has been conducted in adults.^[22]^ Treatment can be divided into three categories: abortive (administered during the prodrome or onset of the attack), prophylactic (given daily to prevent further episodes), and supportive (provided during the attack to alleviate symptoms). The suggested algorithm for management of CVS is shown in Figure 1.^[23]^

During severe bouts of cyclic vomiting, patients require abortive and supportive care which may include admission to the emergency department and/or hospital. The patient we reported was given Ondansetron, proton pump inhibitor, and maintenance of water and electrolyte balance. Stimulation due to light and sound should be minimized, so the lights and curtains of the ward were turned off in order to reduce the stimulation of light and sound. The treatment lasted for 2 to 3 days and was effective, then she recovered and discharged from hospital. Although triptans and benzodiazepines are considered effective abortive therapy,^[24,25]^ our treatment medication did not include them. In our experience, Ondansetron appears to be fully effective if administered early in CVS.

After the patient recovered and discharged, we administrated prophylactic medications, because her severity has reached the moderate to severe disease, which was defined as “>4 episodes per year, severe nausea and vomiting, repeated emergency department visits or hospitalizations, or interference with functioning.”^[10]^ Tricyclic antidepressants (TCAs) are the first-line drugs for the prevention of CVS. In adults, amitriptyline is usually titrated at an incremental dose of 10 mg/week starting at 25 mg, to approximately 100 mg/night. Dry mouth and lethargy are common side effects of TCAs.^[26]^ Our patient was given amitriptyline 25 mg at night, and maximal dose of 50 mg at night; this dose did not reach the recommended dose of the Rome IV criteria, and achieved full remission. The reason for this may be due to the low body weight of this patient which was 44 kg. In addition, olanzapine 1.25 mg was also given to the patient at night; olanzapine acts as neuromodulator agents and is effective in clinical applications in the treatment of nausea and vomiting, especially in the management of chronic functional nausea.^[27]^

## 4 Conclusion

CVS in adults is a highly disabling condition with high utilization of emergency department services, but the lack of awareness in clinicians may delay diagnosis for many years. No specific tests have been identified to discern patients with CVS, and diagnosis is based on typical symptoms and the exclusion of other disorders that are associated with recurrent vomiting. Treatment with amitriptyline has been shown to be effective in alleviating the disease. Increased physician awareness could reduce the delayed diagnosis of CVS. Further research is needed to investigate the epidemiology and potential pathophysiological mechanisms, and more rigorous clinical trials are needed to improve the management of this disease.

## Author contributions

**Data curation:** Cuilan Tang.

**Writing – original draft:** Cuilan Tang.

**Writing – review & editing:** Ning Dai.
